# HGF/c-MET signaling induces glutamine metabolism in MET-amplified head and neck squamous cell carcinoma via MAPK/ERK-dependent induction of GLS-1

**DOI:** 10.1007/s12032-026-03409-0

**Published:** 2026-09-15

**Authors:** Marius Hörner, Florian Mersdorf, Natalie Burkard, Andreas Vollmer, Babak Saravi, Tobias Renner, Julian Volland, Alexander Kübler, Nicolas Schlegel, Stefan Hartmann

**Affiliations:** 1 Department of Oral and Maxillofacial Plastic-Head- and Neck Surgery, University Hospital of Würzburg, Pleicherwall 2, 97070 Würzburg, Germany; 2 Department of General, Visceral, Transplant, Vascular and Pediatric Surgery, University Hospital , Würzburg, Germany; 3 Department of Oral, Maxillofacial and Facial Plastic Surgery, Medical Faculty and University Hospital Düsseldorf, Heinrich-Heine-University Düsseldorf, 40225 Düsseldorf, Germany

**Keywords:** HNSCC, MAPK/ERK, HGF, Glutamine metabolism, MET-signaling

## Abstract

Head and neck squamous cell carcinoma (HNSCC) is a common malignancy characterized by poor survival due to recurrence, metastasis and therapy resistance. In addition to genetic alterations, metabolic reprogramming is a hallmark of HNSCC and contributes to tumor progression and treatment failure. The hepatocyte growth factor (HGF)/c-MET signaling pathway is frequently activated in head and neck squamous cell carcinoma (HNSCC), where it promotes tumor cell proliferation, invasion, and increased glucose metabolism. However, its contribution to the regulation of glutamine metabolism in HNSCC remains largely unexplored. Basal GLS-1 expression was compared between primary human oral keratinocytes (HOK) and HNSCC cell lines with distinct MET status (Detroit 562, FaDu, and SCC-154). Subsequently, HNSCC cell lines were stimulated with HGF, and GLS-1 expression was assessed by quantitative PCR and Western blotting. Functional effects were evaluated using wound-healing assays, enzymatic quantification of extracellular glutamine and glutamate, and Seahorse-based mitochondrial respiration analysis. GLS-1 was silenced using siRNA to determine its functional relevance. In addition, transcriptomic data from the TCGA-HNSC cohort were analyzed to evaluate the clinical association between MET signaling, MAPK/ERK pathway activity, and GLS1 expression. HGF stimulation selectively induced GLS-1 expression in MET-amplified Detroit 562 cells at both mRNA and protein levels, whereas MET wild-type cells remained largely unaffected. Mechanistically, HGF triggered robust ERK1/2 activation, and pharmacological inhibition of c-MET or ERK signaling abrogated GLS-1 induction. Functionally, HGF increased glutamine consumption, glutamate production, and mitochondrial respiratory capacity in a GLS-1–dependent manner, while GLS-1 silencing significantly impaired HGF-induced cell migration and mitochondrial respiration. Importantly, analysis of TCGA-HNSC tumors revealed significant correlations between MET expression, MAPK/ERK pathway activity, and GLS1 expression, supporting the clinical relevance of the identified signaling axis. Together, these findings identify GLS-1 as a downstream effector of HGF/c-MET–MAPK/ERK signaling in MET-amplified HNSCC and link oncogenic signaling to glutamine metabolism and mitochondrial function. The observed associations in TCGA patient tumors further support the clinical relevance of this pathway and suggest that targeting glutaminase or ERK signaling may represent a promising therapeutic strategy in c-MET–driven HNSCC.

**Graphic abstract:**

**Figure:**
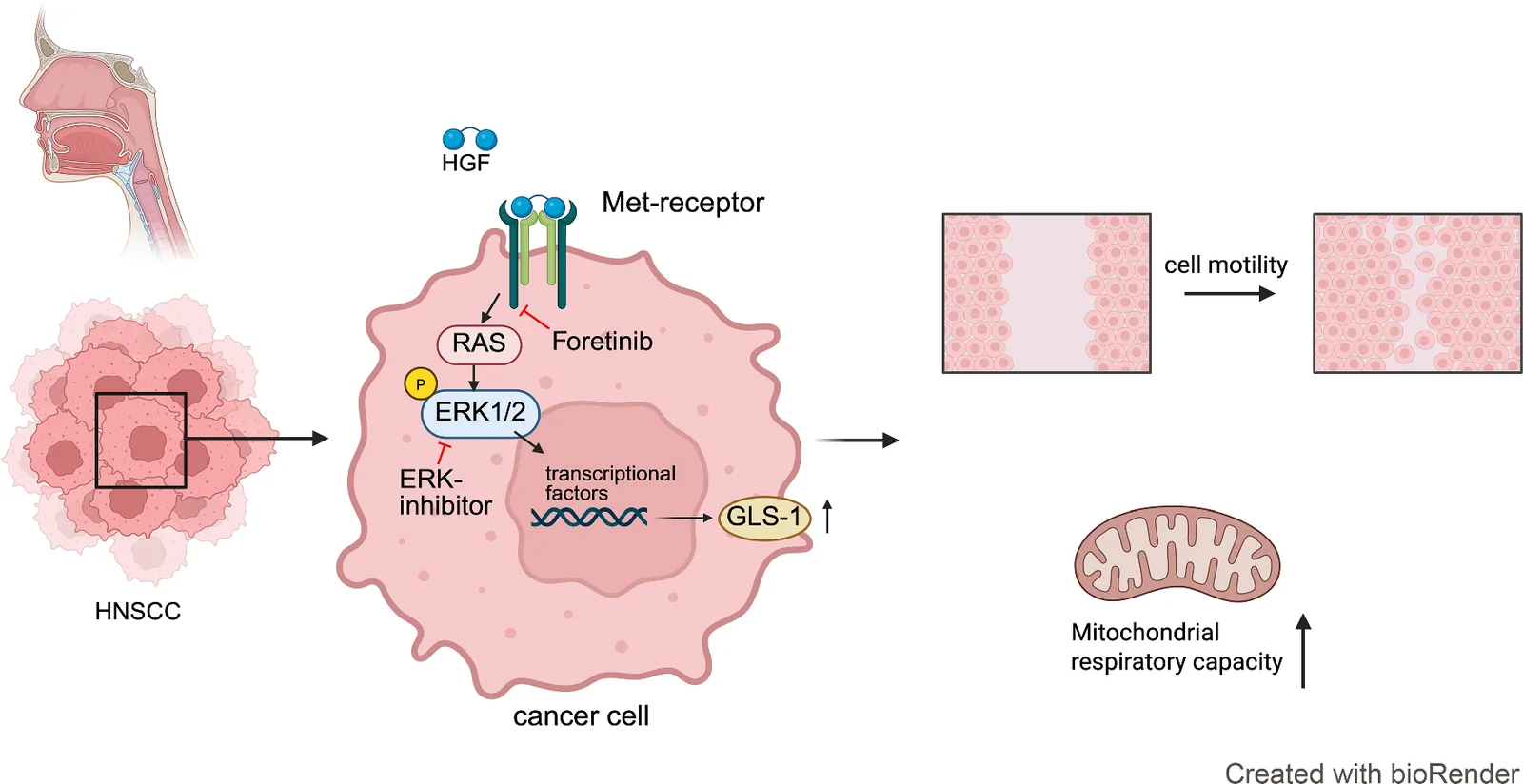

The schematic summarizes the proposed mechanism: HGF binding to the c-MET receptor activates the MAPK/ERK signaling pathway, resulting in transcriptional upregulation of GLS-1. Increased GLS-1 expression enhances glutamine metabolism and mitochondrial oxidative phosphorylation, thereby promoting metabolic reprogramming and increased cell motility in MET-amplified HNSCC cells. Pharmacologic inhibition of c-MET or ERK1/2, as well as GLS-1 silencing, disrupts this signaling axis, attenuating both metabolic activity and HGF-driven migration. Collectively, these findings identify GLS-1 as a critical downstream effector of HGF/c-MET signaling that links oncogenic signaling to metabolic adaptation and tumor progression in HNSCC. Created in BioRender. Schlegel, N. (2026) https://BioRender.com/l8a7a1t

**Supplementary Information:**

The online version contains supplementary material available at https://doi.org/10.1007/s12032-026-03409-0.

## Introduction

Head and neck squamous cell carcinoma (HNSCC) represents the sixth most common cancer worldwide, with nearly 900,000 new cases annually and mortality rates approaching 50% [7, 14]. The high lethality is primarily due to late-stage diagnosis, loco-regional recurrence, and resistance to current therapeutic regimens. Despite advances in surgery, radiotherapy, chemotherapy, and immunotherapy, treatment options remain limited, particularly for patients with recurrent or metastatic disease.

The incidence of oral cancer is closely associated with detrimental oral-related behaviors such as tobacco smoking and excessive alcohol consumption, as well as exposure to human papillomavirus (HPV). Both smoking and alcohol have been linked to altered cellular metabolism and tumorigenesis [14]. Indeed, metabolic reprogramming is a hallmark of cancer and enables tumor cells to adapt to nutrient stress and sustain uncontrolled proliferation [23]. Classical metabolic adaptations include enhanced glycolytic flux, the so-called Warburg effect, as well as the utilization of alternative energy sources such as glutamine through glutaminolysis [9]. Glutamine serves as a critical nutrient for proliferating cancer cells, providing carbon and nitrogen to support anabolic processes including nucleotide, amino acid, and lipid biosynthesis. The rate-limiting enzyme glutaminase 1 (GLS-1) catalyzes the conversion of glutamine to glutamate, fueling the tricarboxylic acid (TCA) cycle and promoting tumor growth. Upregulation of GLS-1 has been reported in several cancers and is associated with aggressive tumor behavior and poor prognosis [30].

In addition to classical risk factors, recent studies have highlighted the role of the tumor microenvironment and immune landscape in HNSCC prognosis. High tertiary lymphoid structure (TLS) gene signatures have been associated with increased immune infiltration and improved clinical outcomes in HNSCC patients, underscoring the relevance of immune-metabolic interactions in disease progression [31]. Furthermore, altered expression of metabolic transporters such as SLC2A3 has been implicated in HNSCC development and immune suppression, suggesting that changes in metabolic regulation can influence both tumor behavior and the immune microenvironment [13].

The hepatocyte growth factor (HGF)/c-MET signaling pathway is a receptor tyrosine kinase axis that plays essential roles in embryonic development, tissue regeneration, and wound healing. In cancer, aberrant activation of this pathway promotes cell proliferation, invasion, angiogenesis, and metastasis. Overexpression of c-MET has been reported in more than 80% of HNSCC cases and correlates with poor prognosis [26]. Moreover, HGF/c-MET signaling contributes to therapeutic resistance, including resistance to EGFR inhibitors such as Cetuximab [29].

While HGF/c-MET signaling has been linked to enhanced glucose metabolism, its impact on glutamine utilization in HNSCC remains poorly understood. Given the central role of glutamine metabolism in tumor progression, elucidating the interaction between HGF/c-MET signaling and GLS-1 dependent metabolic regulation may reveal new therapeutic vulnerabilities. In this study, we investigated whether HGF stimulation modulates GLS-1 expression and glutamine metabolism in HNSCC cells and evaluated the functional and therapeutic implications of GLS-1 inhibition in this context.

## Material and methods

### Cell lines

Among the cell lines listed in Table 1 are established, adherent tumor cells derived from squamous cell carcinomas of the head and neck region. SCC-154 and FaDu originate from the respective primary tumors, while Detroit 562 was derived from a pleural metastasis (ATCC, 2018). In addition, primary Human Oral Keratinocytes (HOK; Cat. No. 2610, ScienCell Research Laboratories, Carlsbad, CA, USA) were included as a non-malignant control. HOK cells were cultured in Oral Keratinocyte Medium (OKM; Cat. No. 2611, ScienCell Research Laboratories) supplemented with Oral Keratinocyte Growth Supplement (OKGS; Cat. No. 2652) and 1% penicillin/streptomycin (Cat. No. 0503) according to the manufacturer's instructions.

**Table 1:** Antibodies, growth factors and inhibitors used in this study. primary and secondary antibodies and their dilutions used for western blots (WB), in vitro (iv) and immunofluorescence (IF)

Antibody	Catalog No	source	Application
Glutaminase−1	#49,363	Cell Signaling Technology, Danvers, USA	WB
Glutaminase−1/GLS-1	#56,750	Cell Signaling Technology, Danvers, USA	WB
β-Actin	#A5316	Sigma Aldrich, St. Louis, USA	WB
Erk1/2 (9107),		Selleckchem Houston, TX, USA	WB
P-Erk1/2 (4370)		Selleckchem Houston, TX, USA	WB
Secondary Ab (HRP)	#7074	Cell Signaling Technology, Danvers, USA	WB
Foretinib	GSK-1363029	Selleckchem (Houston, TX, USA	iv
FR180204	FR 180204	Sigma-Aldrich, St. Louis, MO, USA	iv
Ki-67	ab15580	Abcam, Cambridge, UK	WB
FAK	3284 S	Cell signaling, Danvers, USA	WB
p-FAK	3285 S	Cell signaling, Danvers, USA	WB

All cell cultures were maintained at 37 °C in a humidified atmosphere containing 5% CO₂. The HNSCC cell lines were cultured in MEM alpha (Thermo Fisher Scientific, USA), split twice weekly, and reseeded in fresh medium. Cell lines were routinely tested for mycoplasma contamination during the experiments using the MycoAlert™ PLUS Mycoplasma Detection Kit (Lonza, Oregon, USA). Briefly, 2 mL of cell culture supernatant were transferred into a 1.5 mL microcentrifuge tube and centrifuged for 5 min at 200 × g. Subsequently, 100 µL of the supernatant were transferred into a 96-well microtiter plate, followed by the addition of 100 µL MycoAlert™ PLUS Reagent. After 5 min of incubation, luminescence was measured. Subsequently, 100 µL of MycoAlert™ PLUS Substrate was added to each well, and a second luminescence measurement was performed after 10 min. Control conditions refer to unstimulated cells cultured under identical conditions without HGF treatment.

### siRNA

The cells were cultured in 6-well plates for a period of 24 h prior to transfection as described before [19]. This was achieved by the introduction of human GLS-1- specific siRNA (Thermo Fisher Scientific, USA), with non-target siRNA serving as the control (Abcam, UK). Transfection was initiated once the cells had reached 80% confluence. The transfection was conducted using Lipofectamine Transfection LTX Reagent (Thermo Fisher Scientific, USA), in accordance with the instructions provided by the manufacturer. Based on preliminary experiments, sufficient knockdown GLS-1 was observed after 24 h (Western blots), and all subsequent experiments were conducted at this time point.

### Western blot

For Western blotting, Detroit 562, FaDu, and SCC-154 cells were lysed in RIPA buffer supplemented with protease and phosphatase inhibitors, as previously described in [15]. Protein lysates were homogenized and quantified using the BCA Protein Assay Kit (Thermo Fisher Scientific, Massachusetts, USA). Equal amounts of protein were separated by SDS–PAGE and transferred onto nitrocellulose membranes. Membranes were blocked and incubated overnight at 4 °C with the respective primary antibodies (Table 1), followed by incubation with HRP-conjugated secondary antibodies for 1 h at room temperature. Protein bands were visualized using the SuperSignal™ West Pico PLUS Chemiluminescent Substrate (Thermo Fisher Scientific, Massachusetts, USA). Densitometric analysis and normalization to β-actin were performed using ImageLab software (Bio-Rad, Hercules, CA, USA).

### Scratch assay

Detroit 562 cell monolayers were cultured in 6-well plates until reaching confluence. Linear wounds were generated using a 10 μL sterile pipette tip, as previously described in [10]. All scratches were performed by the same blinded investigator to ensure reproducibility, followed by replacement of the culture medium. Images of the wound area were captured at 0, 24 and 48 post-scratch. Wound closure was quantified using ImageJ software by measuring the remaining wound area relative to the initial (t = 0) area. Cells were treated with HGF (50 ng/mL), Foretinib (100 nM), FR180204 (10 µM), or the respective combinations as indicated (Table 2).

**Table 2:** Table 2 lists the cell lines used in this study, including their tissue of origin and supplier

Name	Tissue origin	supplier
SCC-154	Tongue	German Collection of Microorganisms and Cell Cultures (DSMZ)
FaDu	Pharynx	Head and Neck Cancer Panel (TCP-1012), American Type Culture Collection (ATCC), Manassas, Virginia, USA
Detroit 562	Pharyngeal metastases, pleura	American Type Culture Collection (ATCC), Manassas, Virginia, USA
Human oral keratinocytes	Oral mucosa	ScienCell Research Laboratories, Carlsbad, CA, USA

### qPCR

Total RNA was extracted from Detroit 562 cells using TRIzol™ reagent (Thermo Fisher Scientific, Waltham, MA, USA) according to the manufacturer’s instructions. Complementary DNA (cDNA) was synthesized from 1 µg of total RNA using the iScript™ cDNA Synthesis Kit (Bio-Rad, Munich, Germany). Quantitative PCR was performed with MESA GREEN qPCR MasterMix Plus for SYBR® Assay (Eurogentec, Cologne, Germany) on a CFX96 Touch Real-Time PCR Detection System (Bio-Rad, Munich, Germany). Relative gene expression was calculated using the ΔΔCt method, with β-actin as the reference gene. Reactions were run in duplicate at an annealing temperature of 60 °C, with primer concentrations of 5 µM. Primer sequences are listed in Table 3.

**Table 3:** Table 3 lists the qPCR primers

Prime gene	Supplier
Hs_ACTB_2_SG	Qiagen, Venlo, Netherlands; Order No.: 3507814
Hs_GLS_1_SG	Qiagen, Venlo, Netherlands Order No.: 3507814

### Enzymatic quantification of glutamate and glutamine in culture supernatants

Glutamate levels in cell culture supernatants were quantified using a commercially available enzymatic Glutamate Assay Kit (Sigma-Aldrich, MAK339, St. Louis, MO, USA) according to the manufacturer’s instructions. Briefly, cell culture supernatants were collected, cleared by centrifugation, and diluted as required. Samples and standards were incubated with the reaction mix, and absorbance was measured at the indicated wavelength using a microplate reader (Tecan, Männedorf, Switzerland). Glutamate concentrations were calculated from a standard curve and normalized to total cellular protein content.

Glutamine levels in cell culture supernatants were determined using a Glutamine Assay Kit (Sigma-Aldrich, MAK438, St. Louis, MO, USA) according to the manufacturer’s instructions. Sample preparation and data acquisition were performed analogously, and glutamine concentrations were quantified using a standard curve and normalized to total cellular protein content.

### Mitochondrial respiration (Seahorse)

Mitochondrial respiration was assessed using the Seahorse XF Cell Mito Stress Test as previously described [18]. Oxygen consumption rate (OCR) was measured in Detroit 562 and FaDu cells using a Seahorse XF96 Analyzer under basal conditions and following sequential injection of oligomycin, the mitochondrial uncoupler BAM15 (as an alternative to FCCP), and rotenone/antimycin A.

Cells were seeded into Seahorse XF96 microplates, transfected, and stimulated with HGF (50 ng/mL) or left untreated. Prior to measurement, culture medium was replaced with Seahorse XF assay medium supplemented with glucose, pyruvate, and glutamine, and cells were incubated at 37 °C in a non-CO₂ environment.

Oligomycin (1.5 µM), BAM15 (3 µM), and rotenone/antimycin A (0.5 µM) were injected sequentially to determine basal and maximal mitochondrial respiration as well as non-mitochondrial respiration. Data were normalized to cell number using crystal violet staining.

### TCGA-HNSC transcriptomic analysis

To assess the clinical relevance of the observed in vitro findings, publicly available RNA-seq data from The Cancer Genome Atlas Head and Neck Squamous Cell Carcinoma cohort (TCGA-HNSC) were analyzed. Transcripts per million (TPM)-normalized gene expression data were obtained for 522 primary tumor samples. Expression values were log2-transformed (TPM + 1) prior to analysis. Spearman rank correlation analyses were performed to evaluate the association between MET, HGF, and GLS1 (GLS) expression. Since ERK phosphorylation cannot be directly assessed from transcriptomic data, MAPK/ERK pathway activity was estimated using a composite transcriptional activity score. This score was calculated as the mean z-score of ten established ERK-responsive target genes (DUSP6, ETV1, ETV4, ETV5, SPRY2, SPRY4, FOSL1, MYC, EGR1, CCND1), which are well-characterized transcriptional readouts of MAPK/ERK signaling. Patients were stratified into MET-high and MET-low groups based on median MET expression; differences in gene expression between groups were assessed using the Mann–Whitney U test. All statistical analyses were performed using Python (SciPy v1.11); p ≤ 0.05 was considered statistically significant. Spearman correlation matrices were computed across all genes of interest and visualized as hierarchically clustered heatmaps.

### Statistics

Statistical analyses were performed using Prism (GraphPad Software, La Jolla, CA, USA). Data are presented as mean ± SEM, and p* ≤ *0.05* was considered statistically significant. Normality was assessed using the Shapiro–Wilk test. Two-group comparisons were conducted using paired Student’s t-tests or Mann–Whitney U tests, as appropriate. Multiple-group analyses were performed using one-way ANOVA with Tukey’s or Šídák’s multiple comparisons test or Bonferroni correction; non-parametric data were analyzed using the Kruskal–Wallis test.

## Results

### HGF/c-MET signaling induces GLS-1 expression in Met-amplified HNSCC cells

**Fig. 1:**
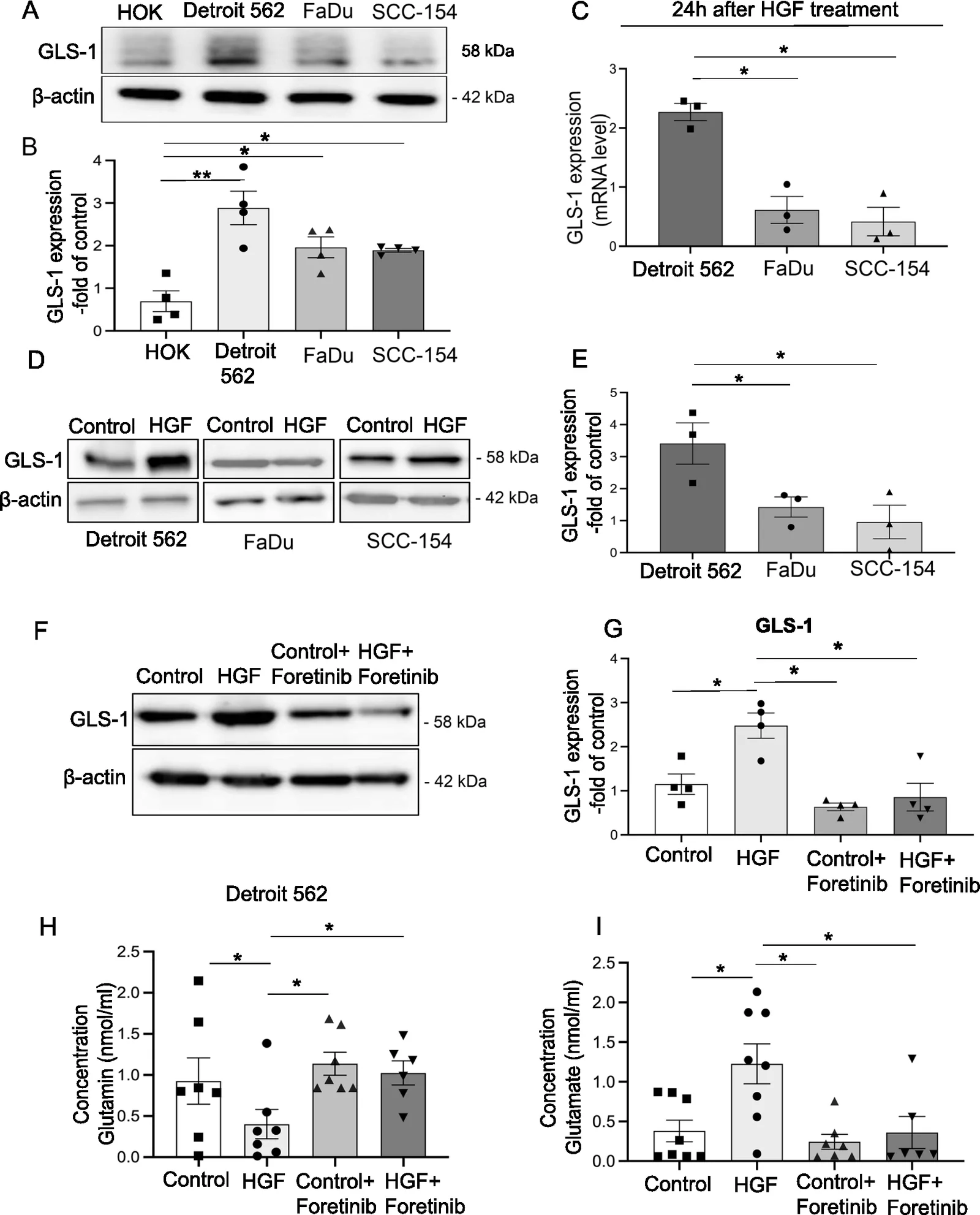
Basal GLS-1 expression and HGF-induced regulation of glutamine metabolism in HNSCC cells (**A**) Representative Western blot analysis showing basal GLS-1 protein expression in primary human oral keratinocytes (HOK) and HNSCC cell lines (Detroit 562, FaDu, SCC-154). β-actin served as a loading control. (**B**) Densitometric quantification of basal GLS-1 protein expression relative to β-actin. Data are presented as fold change relative to HOK. (**C**) Quantitative RT–PCR analysis of GLS-1 mRNA expression in HNSCC cell lines (Detroit 562, FaDu, SCC-154) following HGF stimulation (50 ng/mL, 24 h). Data are presented as mean ± SEM from three independent experiments. (**D**) Representative Western blot analysis showing GLS-1 protein expression in Detroit 562, FaDu, and SCC-154 cells following HGF stimulation. β-actin served as a loading control. (**E**) Densitometric quantification of GLS-1 protein expression relative to β-actin and normalized to the respective untreated controls. (**F**) Representative Western blot analysis of GLS-1 protein expression in Detroit 562 cells treated with HGF (50 ng/mL, 24 h) in the presence or absence of the c-MET inhibitor Foretinib (100 nM, 2 h pre-treatment).(**G**) Densitometric quantification of GLS-1 protein expression relative to β-actin. HGF-induced GLS-1 upregulation was abolished by c-MET inhibition with Foretinib. (**H**) Extracellular glutamine concentrations (nmol/mL) in control cells, HGF-treated cells, and cells treated with Foretinib in the absence or presence of HGF. (**I**) Extracellular glutamate concentrations (nmol/mL) under the same experimental conditions. Individual data points represent biological replicates, and bars indicate mean ± SEM from at least three independent experiments. Statistical analyses were performed using one-way ANOVA followed by Tukey's multiple-comparisons test. *p ≤ 0.05

To determine whether GLS-1 is upregulated in HNSCC cells compared with non-malignant oral epithelium, we first analyzed basal GLS-1 protein expression in primary human oral keratinocytes (HOK) and three representative HNSCC cell lines. As shown in Fig. 1A–B, GLS-1 expression was consistently higher in all HNSCC cell lines than in HOK cells, with the strongest basal expression observed in the MET-amplified Detroit 562 cell line. These findings indicate that GLS-1 is upregulated in HNSCC cells and provide the rationale for investigating the regulation of GLS-1 by HGF/c-MET signaling.

Previous studies have demonstrated that stimulation of the c-MET signaling pathway by HGF enhances the expression of key glycolytic enzymes in HNSCC [4]. However, whether HGF/c-MET signaling also affects glutamine metabolism has remained unclear. We therefore hypothesized that HGF stimulation modulates the glutamine metabolic program of HNSCC cells through c-MET dependent mechanisms.

To test this, we analyzed the expression of glutaminase 1 (GLS-1), the rate-limiting enzyme in glutamine metabolism, in three representative HNSCC cell lines with distinct MET status, following stimulation with HGF: Detroit 562 (MET-amplified), and FaDu and SCC-154 (both MET wild-type).

Cells were treated with recombinant HGF (50 ng/mL) for 24 h, and GLS-1 expression was quantified by qPCR (Fig. 1C) and Western blot analysis (Fig. 1D).

HGF stimulation markedly increased GLS-1 mRNA levels in MET-amplified Detroit 562 cells (3.41 ± 0.33-fold vs. control), whereas no significant changes were observed in FaDu (0.42 ± 0.18) or SCC-154 cells (0.95 ± 0.35; Fig. 1C).

To determine whether this regulation also occurred at the protein level, GLS-1 expression was examined by Western blot analysis (Fig. 1D). Consistent with the qPCR results, HGF treatment led to a robust increase in GLS-1 protein levels in MET-amplified Detroit 562 cells, showing several-fold higher expression compared to untreated controls (Fig. 1F). In contrast, FaDu and SCC-154 cells showed no significant changes in GLS-1 expression under identical conditions. To confirm that GLS-1 induction depends on c-MET activity, Detroit 562 cells were treated with the selective c-MET inhibitor Foretinib prior to HGF stimulation (Fig. 1F).

HGF stimulation markedly increased GLS-1 protein levels in Detroit 562 cells, whereas co-treatment with the c-MET inhibitor Foretinib abrogated this effect, restoring GLS-1 expression to baseline levels. Treatment with Foretinib alone had no detectable impact on GLS-1 expression (Fig. 1F-G).

Given that HGF/c-MET signaling selectively induced GLS-1 expression in MET-amplified Detroit 562 cells, we next investigated whether this transcriptional and translational upregulation was associated with functional alterations in glutamine metabolism. To this end, extracellular glutamine and glutamate levels were quantified in cell culture supernatants following 24 h of HGF stimulation and pharmacological inhibition of c-MET signaling.

As shown in Fig. 1H, HGF treatment resulted in a significant reduction of extracellular glutamine concentrations compared with untreated controls, consistent with increased glutamine consumption. Importantly, inhibition of c-MET signaling by Foretinib largely prevented this decrease, restoring glutamine levels to those observed under control conditions.

In parallel, extracellular glutamate concentrations were markedly increased upon HGF stimulation (Fig. 1I). This HGF-induced accumulation of glutamate was abrogated by Foretinib, both under basal conditions and following HGF treatment, indicating that enhanced glutamate production depends on intact c-MET activity.

Together, these data demonstrate that HGF/c-MET–dependent induction of GLS-1 is accompanied by a metabolic shift toward increased glutamine utilization and glutamate production in MET-amplified HNSCC cells. These functional metabolic changes provide mechanistic support for the observed regulation of GLS-1 expression and further substantiate a role for HGF/c-MET signaling in reprogramming glutamine metabolism.

Collectively, these findings identify GLS-1 as a downstream effector of HGF/c-MET signaling in MET-amplified HNSCC cells and establish a mechanistic connection between c-MET activation and glutamine metabolism as a key component of tumor-associated metabolic reprogramming.

### HGF/c-MET signaling induces GLS-1 expression via MAPK/ERK activation

**Fig. 2:**
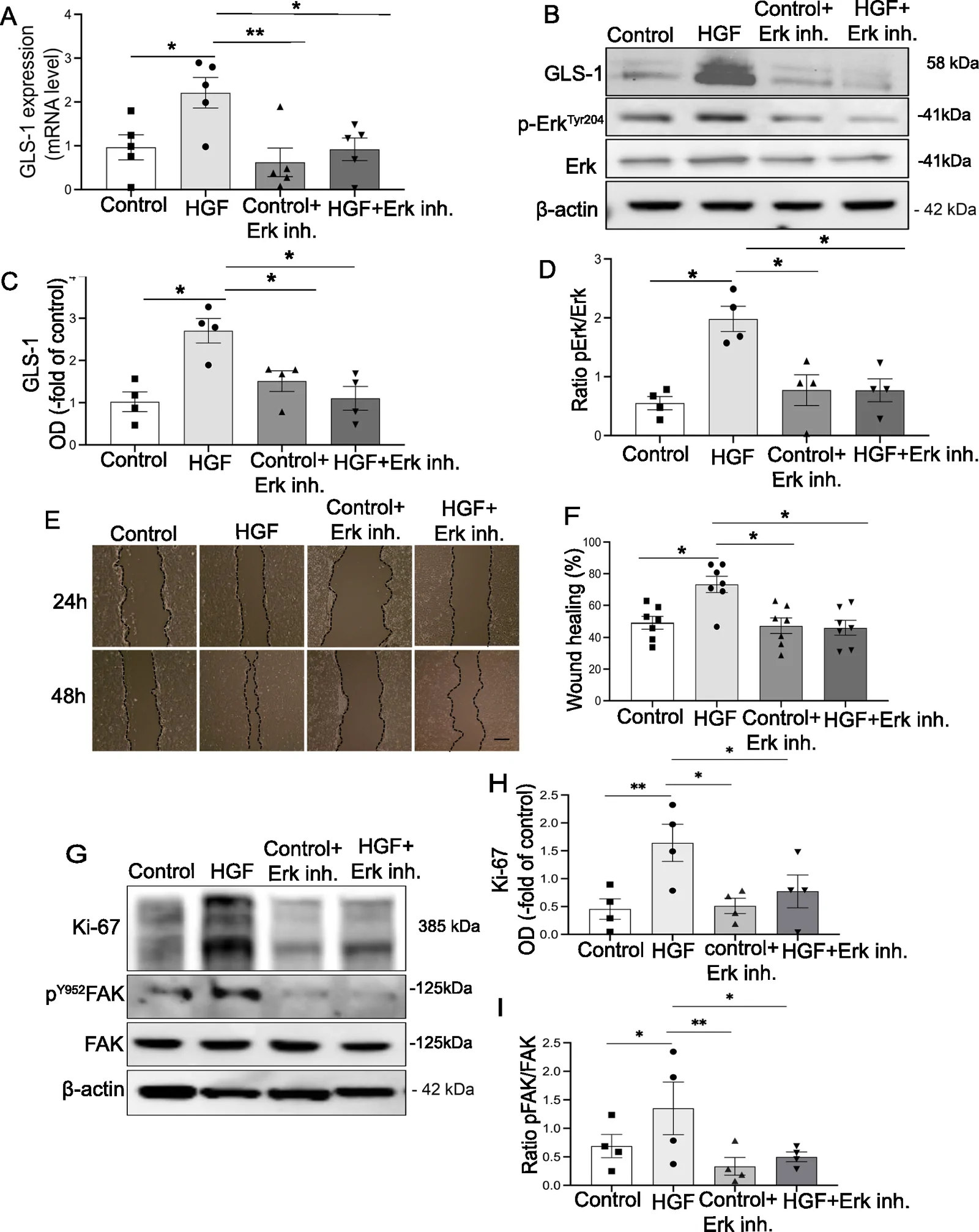
MAPK/ERK signaling mediates HGF-induced GLS-1 upregulation and cell migration (**A**) Quantitative RT–PCR analysis of GLS-1 mRNA expression in Detroit 562 cells treated with HGF (50 ng/mL, 24 h) in the presence or absence of the selective ERK1/2 inhibitor FR180204 (10 µM, 2 h pre-treatment). Data are shown as mean ± SEM from three independent experiments. (**B**) Representative Western blot analysis of GLS-1, phosphorylated ERK1/2 (p-ERK), and total ERK protein levels following HGF stimulation and ERK inhibition. β-actin served as a loading control. (**C**) Densitometric quantification of GLS-1 protein expression relative to β-actin normalized to control. (**D**) Quantification of ERK activation shown as p-ERK/ERK ratio relative to control. (**E**) Representative wound-healing assay images (scale bar, 200 µm) showing enhanced migration after HGF stimulation and suppression of this effect by ERK inhibition at 24 h and 48 h. (**F**) Quantification of wound closure expressed as percentage of the initial wound area. (**G**) Representative Western blot analysis of Ki-67, phosphorylated FAK and total FAK protein levels following HGF stimulation and ERK inhibition. β-actin served as a loading control (**H**) Densitometric quantification of Ki-67 protein expression relative to β-actin normalized to control. (**I**) Quantification of FAK activation shown as p-FAK/FAK ratio relative to control. Data are shown as mean ± SEM and were analysed by one-way ANOVA followed by Tukey’s post hoc test; p* ≤ 0.05

To elucidate the signaling mechanisms underlying HGF-induced GLS-1 upregulation, we focused first on the MAPK/ERK pathway. This choice was based on our previous glucose-metabolism studies, in which neither PI3K/AKT nor JAK/STAT signaling contributed to the metabolic switch, making MAPK/ERK the most plausible candidate for further investigation in MET-amplified Detroit 562 cells [4]. Quantitative PCR analysis showed that HGF stimulation led to a marked increase in GLS-1 mRNA levels, whereas co-treatment with the selective ERK1/2 inhibitor FR180204 completely abolished this effect (Fig. 2A).

Western blot analysis confirmed that HGF induced a strong phosphorylation of ERK1/2^Tyr204^ and concomitantly elevated GLS-1 protein levels (Fig. 2B). Densitometric quantification revealed a significant increase in GLS-1 protein expression and p-ERK/ERK ratios following HGF stimulation, both of which were reduced to baseline upon ERK inhibition (Fig. 2C). Treatment with the inhibitor alone had no detectable effect on GLS-1 or ERK phosphorylation.

Functionally, HGF stimulation significantly enhanced cell motility, as evidenced by accelerated wound closure of 65.42 ± 5.13% after 24 h and 89.67 ± 3.25% after 48 h, compared to 40.28 ± 4.31% and 70.54 ± 6.02% in control cells. In contrast, pharmacological inhibition of ERK1/2 with FR180204 abrogated this pro-migratory effect, reducing wound closure to near baseline levels (Fig. 2E–F).

It is well established that epithelial regeneration and tumor progression involve a coordinated interplay between cell proliferation and motility-related processes [22]. To further delineate the contribution of HGF/c-MET-dependent migratory signaling, we analyzed phosphorylation of focal adhesion kinase (FAK) as a surrogate marker of migratory activity [17]. Western blot analyses revealed a significant increase in FAK phosphorylation following HGF stimulation, which was attenuated upon inhibition of c-MET signaling (Fig. 2 G/I).

Collectively, these findings demonstrate that HGF/c-MET signaling activates the MAPK/ERK pathway, which in turn drives GLS-1 expression and promotes a migratory and proliferative phenotype in MET-amplified HNSCC cells. We cannot exclude additional ERK-independent mechanisms contributing to GLS-1 regulation.

### GLS-1 is required for HGF-Induced cell motility and mitochondrial respiration

**Fig. 3:**
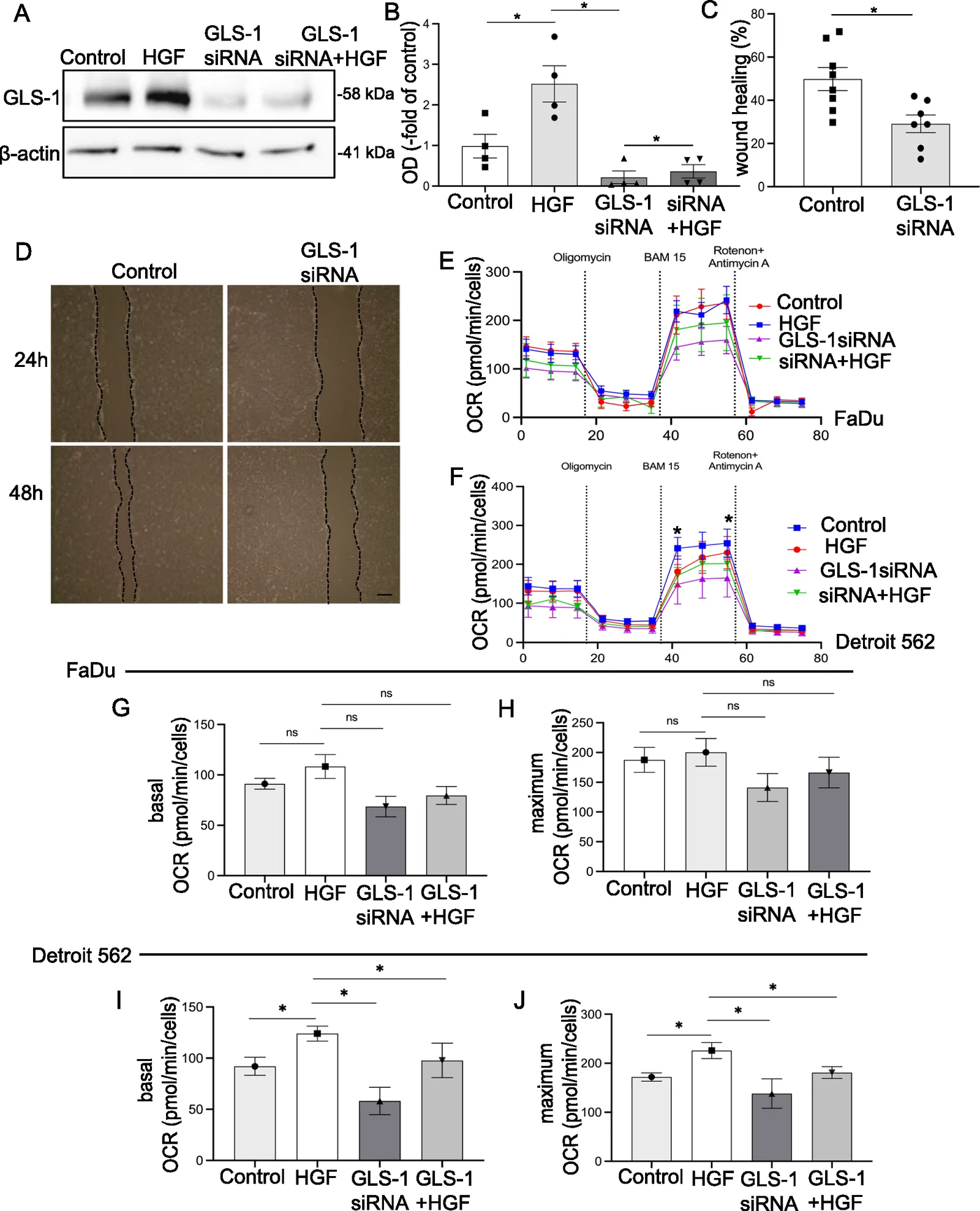
GLS-1 knockdown suppresses HGF-driven GLS-1 expression and cell motility in 562 cells (**A**) Representative Western blot analysis showing GLS-1 protein expression in Detroit 562 cells following HGF stimulation (50 ng/mL, 24 h) with or without GLS-1 knockdown by siRNA, β-actin served as a loading control. (**B**) Densitometric quantification of GLS-1 expression normalized to β-actin and presented as fold change relative to control. HGF treatment markedly increased GLS-1 protein levels, whereas GLS-1 siRNA silencing effectively suppressed both basal and HGF-induced GLS-1 expression. (**C**) Quantification of wound closure relative to the initial scratch area (t = 0). GLS-1 knockdown significantly impaired wound healing, indicating reduced migratory capacity. (**D**) Representative images of wound-healing assays at 24 h and 48 h post-scratch in control and GLS-1 siRNA-transfected Detroit 562 cells. Scale bar, 200 µm. (**E**–**F**) Representative Seahorse XF Mito Stress Test profiles showing oxygen consumption rate (OCR) in FaDu (**E**) and Detroit 562 (**F**) cells following GLS-1 siRNA transfection and ± HGF stimulation. Oligomycin, BAM15, and rotenone/antimycin A were sequentially injected as indicated. Quantification of basal (**G**) and maximal (**H**) mitochondrial respiration in FaDu cells derived from Seahorse measurements. (**I**–**J**) Quantification of basal (**I**) and maximal (**J**) mitochondrial respiration in MET-amplified Detroit 562 cells. HGF increased both basal and maximal respiration in a GLS-1-dependent manner. Data are shown as mean ± SEM and were analysed by one-way ANOVA followed by Tukey’s post hoc test; (p* ≤ 0.05, p** ≤ 0.01, p*** ≤ 0.001)

To evaluate the functional relevance of GLS-1 induction, we next assessed the effects of GLS-1 depletion on cell proliferation and migration. GLS-1 expression was efficiently silenced by siRNA in Detroit 562 cells, as confirmed by Western blot analysis showing a marked reduction of GLS-1 protein levels compared with non-targeting controls (Fig. 3A). Upon HGF stimulation, GLS-1 expression was strongly upregulated in control cells, whereas this effect was completely abolished in GLS-1 siRNA-transfected cells, indicating that HGF-induced GLS-1 elevation depends on intact GLS-1 expression (Fig. 3A–B).

Following GLS-1 knockdown, wound-healing assays were performed to determine migratory capacity. GLS-1 depletion significantly delayed wound closure in Detroit 562 cells, indicating impaired migratory behavior (Fig. 3C-D).

To determine whether GLS-1 silencing is associated with functional metabolic changes, mitochondrial respiration was assessed using Seahorse extracellular flux analysis. Oxygen consumption rate (OCR) was measured under basal conditions and following sequential injection of oligomycin, the mitochondrial uncoupler BAM15, and rotenone/antimycin A.

In MET-amplified Detroit 562 cells, HGF stimulation significantly increased both basal and maximal mitochondrial respiration compared with unstimulated controls (Fig. 3F, I–J). GLS-1 silencing by siRNA markedly reduced basal and maximal OCR and abrogated the HGF-induced increase in mitochondrial respiration, indicating that HGF-driven enhancement of oxidative metabolism is dependent on intact GLS-1 expression in this cellular context.

In contrast, MET wild-type FaDu cells exhibited no significant increase in basal or maximal mitochondrial respiration upon HGF stimulation (Fig. 3E, G–H). GLS-1 silencing resulted in a reduction of OCR; however, this effect was largely independent of HGF treatment, suggesting that mitochondrial respiration in MET wild-type cells is less responsive to HGF/c-MET signaling.

Collectively, these data demonstrate that HGF/c-MET signaling enhances mitochondrial respiratory activity in a GLS-1–dependent manner specifically in MET-amplified HNSCC cells, providing functional metabolic evidence that complements the observed regulation of GLS-1 expression and downstream MAPK/ERK signaling. These findings may be particularly relevant for MET-amplified HNSCC subsets, in which metabolic vulnerabilities could be therapeutically exploited.

### Clinical validation: MET and MAPK/ERK pathway activity correlate with GLS1 expression in TCGA-HNSC patients

To determine whether the in vitro association between HGF/c-MET signaling and GLS-1 expression is reflected in clinical tumor specimens, we analyzed RNA-seq data from 522 primary HNSCC tumors in the TCGA-HNSC cohort.

**Fig. 4:**
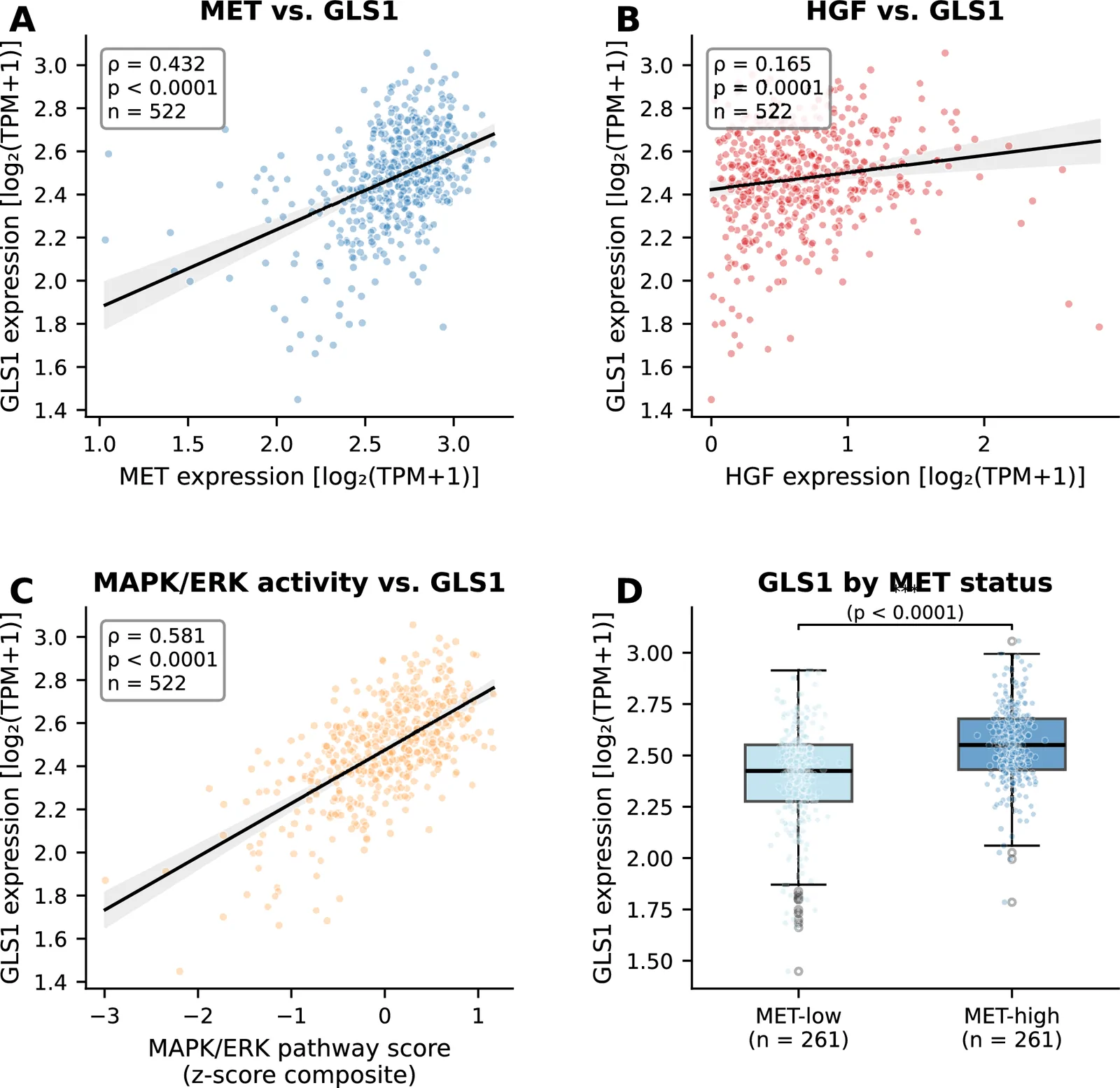
MET expression and MAPK/ERK pathway activity correlate with GLS1 expression in TCGA-HNSC patient tumors (n = 522). (**A**) Spearman correlation between MET and GLS1 mRNA expression. (B) Spearman correlation between HGF and GLS1 mRNA expression. (**C**) Spearman correlation between a composite MAPK/ERK transcriptional activity score (mean z-score of DUSP6, ETV1, ETV4, ETV5, SPRY2, SPRY4, FOSL1, MYC, EGR1, CCND1) and GLS1 expression. (**D**) Comparison of GLS1 expression between MET-high (n = 261) and MET-low (n = 261) patient groups, stratified by median MET expression. All expression values are shown as log2(TPM + 1). Scatter plots include linear regression lines with 95% confidence intervals. Spearman correlation coefficients (ρ) and p-values are indicated. Box plots display median, interquartile range, and individual data points; significance was assessed by Mann–Whitney U test. ***p < 0.001

Spearman correlation analysis revealed a significant positive correlation between MET and GLS1 expression (ρ = 0.432, p < 0.0001; Fig. 4A). A weaker but statistically significant correlation was also observed between HGF and GLS1 (ρ = 0.165, p = 0.0001; Fig. 4B), consistent with the notion that ligand availability alone does not fully determine pathway activation in the absence of receptor amplification. Notably, the strongest correlation was found between a composite MAPK/ERK transcriptional activity score—calculated from ten established ERK target genes—and GLS1 expression (ρ = 0.581, p < 0.0001; Fig. 4C), supporting the mechanistic link between MAPK/ERK signaling and GLS-1 regulation identified in our in vitro experiments.

To further evaluate the relationship between MET expression levels and glutamine metabolism-associated genes, patients were stratified into MET-high and MET-low groups by median MET expression. MET-high tumors exhibited significantly higher GLS1 expression compared with MET-low tumors (p < 0.0001; Fig. 4D). This difference extended to additional MAPK/ERK targets including DUSP6, MYC, and FOSL1, as well as the glutamine transporter SLC1A5 (ASCT2), all of which were significantly upregulated in MET-high tumors (Supplementary Fig. S1). In contrast, GLS2 expression did not differ significantly between MET-high and MET-low groups (p = 0.089), suggesting that the metabolic rewiring is specific to GLS1-dependent glutaminolysis.

Hierarchical clustering of Spearman correlation coefficients across all 20 analyzed genes confirmed that MET, GLS1, MAPK1 (ERK2), and several ERK target genes formed a co-expression cluster, whereas GLS2 clustered separately (Supplementary Fig. S2). These findings provide independent clinical evidence that MET expression and MAPK/ERK pathway activity are associated with GLS1 expression in HNSCC patient tumors, corroborating the in vitro mechanistic data presented in this study.

## Discussion

Head and neck squamous cell carcinoma (HNSCC) remains a challenging malignancy with poor prognosis due to frequent recurrence and therapy resistance. Beyond genetic alterations, recent evidence highlights metabolic reprogramming as a key driver of tumor progression and immune escape. Previous reports have shown that HGF enhances glucose metabolism via glycolytic reprogramming in HNSCC [4]. Our data extend this concept by demonstrating that c-MET activation also drives glutamine metabolism through GLS-1 induction. Such dual control of glucose and glutamine pathways highlights a broader metabolic plasticity of HNSCC cells under HGF stimulation. In line with this, other oncogenic signaling axes such as the PGE₂/PTEN pathway have also been shown to modulate tumor metabolism and growth in HNSCC [21], underscoring the central role of metabolic reprogramming in disease progression.

HGF stimulation markedly upregulated GLS-1 expression, most prominently in MET-amplified Detroit 562 cells, as demonstrated by qPCR and Western blot analysis. Importantly, our observation that basal GLS-1 expression was consistently higher in HNSCC cell lines than in primary human oral keratinocytes is in line with previous reports describing GLS1 overexpression in HNSCC [3, 32].

To elucidate the underlying signaling mechanism, we examined major c-MET downstream pathways. HGF treatment induced robust phosphorylation of ERK1/2 in MET-amplified Detroit 562 cells. Pharmacological inhibition of c-MET with Foretinib or blockade of ERK1/2 with FR180204 abrogated ERK1/2 phosphorylation and prevented the HGF-induced increase in GLS-1 protein levels. Moreover, GLS-1 silencing by siRNA significantly reduced cell motility in wound-healing assays.

These findings demonstrate that HGF/c-MET–driven GLS-1 upregulation is mediated via activation of the MAPK/ERK pathway. The MAPK/ERK cascade represents a central downstream effector of receptor tyrosine kinase signaling, integrating mitogenic and metabolic cues to regulate transcriptional programmes that support cell growth and survival. In addition to MAPK/ERK signaling, dysregulation of PI3K/AKT activity has been associated with enhanced tumor cell motility and proliferation in HNSCC, indicating that balanced regulation of these pathways is critical for controlling oncogenic progression. Together, these observations highlight the coordinated contribution of distinct downstream signaling branches in mediating receptor tyrosine kinase-dependent tumor adaptation [11, 20]. Through activation of transcription factors such as c-Myc, ERK signaling can promote the expression of metabolic enzymes including GLS-1, thereby coupling oncogenic signaling to glutamine metabolism. Functionally, GLS-1 knockdown by siRNA reduced cell proliferation and significantly impaired wound closure, indicating diminished proliferative and migratory capacity.

Our data demonstrate that HGF/c-MET signaling promotes GLS-1-dependent glutamine utilization and that combined inhibition of GLS-1 and c-MET may have synergistic effects on tumor progression, representing a promising future therapeutic strategy particularly in advanced or metastatic HNSCC.

The present work relies exclusively on 2D cell culture models, with mechanistic analyses largely centered on the MET-amplified Detroit 562 cell line. Accordingly, our findings should be interpreted as context-dependent, reflecting a metabolic vulnerability associated with MET amplification rather than a general feature of all HNSCC. Validation in additional MET-amplified models, as well as in 3D spheroids, patient-derived organoids, and in vivo systems, will be essential to assess the broader relevance of the HGF/c-MET-GLS-1 axis.

Moreover, while our data suggest that targeting c-MET or GLS-1 may impair HGF-driven metabolic and migratory phenotypes, translational implications should be interpreted with caution, as combinatorial treatment strategies and in vivo efficacy were not addressed in the current study.

Importantly, analysis of 522 primary HNSCC tumors from the TCGA-HNSC cohort confirmed a significant positive correlation between MET expression, MAPK/ERK transcriptional activity, and GLS1 expression at the patient level, providing clinical support for the in vitro mechanistic findings. The observation that GLS1 was selectively elevated in MET-high tumors, whereas GLS2 was not, further underscores the specificity of GLS1 as a downstream effector of c-MET signaling in HNSCC.

Among the molecular pathways implicated in HNSCC progression, the HGF/c-MET axis has attracted particular attention. This pathway promotes proliferation, migration, invasion, and metastasis [1], and clinical data link HGF and c-MET overexpression to reduced survival in HNSCC patients [28]. HGF stimulation has further been shown to increase PD-L1 expression in renal carcinoma [2]. A similar association was demonstrated in HNSCC in previous work from our group [5].

In hepatocellular carcinoma, HGF was also shown to promote glutaminolysis through activation of GLS-1 [12], suggesting a broader role in metabolic reprogramming.

The pronounced upregulation of GLS-1 following HGF stimulation, particularly in MET-amplified Detroit 562 cells, underscores the metabolic relevance of the HGF/c-MET signaling axis in HNSCC. Detroit 562 cells originate from a metastasis of a pharyngeal carcinoma, suggesting that c-MET–driven glutamine metabolism may represent a metabolic adaptation associated with the metastatic phenotype [4]. Our findings expand this concept by linking c-MET activation to glutamine metabolism via upregulation of GLS-1, the rate-limiting enzyme in glutaminolysis. Interestingly, earlier reports have shown that c-MET activation can also induce PD-L1 expression in metastatic HNSCC, suggesting a broader role of this pathway at the intersection of oncogenic signaling, metabolic plasticity, and immune evasion [5].

Glutamine availability is increasingly recognized as a critical determinant of antitumor immune responses. Although immune components were not included in our experimental models, the observed link between HGF/c-MET signaling and glutamine metabolism raises the possibility that this pathway may indirectly influence the tumor immune microenvironment, an aspect that warrants dedicated investigation in immunocompetent models [9, 27].

The emerging concept that c-MET coordinates tumor metabolic reprogramming and immune modulation offers a compelling rationale for combinatorial therapeutic strategies in advanced HNSCC [24]. In the context of immunometabolism, metabolic rewiring of tumor cells can profoundly influence the immune microenvironment, affecting T-cell function and antitumor immunity [34]. This is consistent with clinical observations linking c-MET overexpression to advanced tumor stage and metastatic progression [6, 28].

The therapeutic implications of these findings are notable. While single-agent c-MET inhibitors such as Foretinib or Tivantinib have demonstrated limited efficacy in HNSCC [16, 25], our data suggest that dual targeting of c-MET and GLS-1 may circumvent compensatory signaling and synergistic antitumor effects. Of particular relevance in this context is Amivantamab, a bispecific EGFR/MET antibody currently approved for NSCLC and now being evaluated in HNSCC. Early results from the OrigAMI-4 trial indicate promising activity in patients progressing after checkpoint inhibition and chemotherapy, supporting the concept that combined inhibition of EGFR/MET signaling may enhance therapeutic responsiveness [8]. Together with our findings, these observations highlight a potential rationale for therapeutic strategies integrating MET blockade with metabolic targeting through GLS-1 inhibition. Next-generation glutaminase inhibitors such as CB-839 have demonstrated activity in several malignancies, including triple-negative breast cancer, renal cell carcinoma, and hematological cancers [33]. Combination strategies targeting both c-MET and GLS-1 therefore represent a promising avenue for translational research in HNSCC.

While we demonstrate robust regulation of GLS-1 expression and associated extracellular changes in glutamine and glutamate levels, direct measurements of intracellular metabolic flux were not performed. Thus, although the reciprocal regulation of extracellular glutamine and glutamate is consistent with enhanced glutaminolytic activity, future studies employing isotope-tracing or comprehensive metabolomic analyses will be required to directly quantify glutamine flux and pathway utilization.

In summary, our findings suggest a mechanistic connection between oncogenic c-MET signaling and glutamine metabolism in HNSCC. Through ERK1/2 activation, HGF/c-MET induces GLS-1 expression and cellular motility in a Met-amplified HNSCC cell model. The identification of this pathway expands the functional spectrum of c-MET beyond its classical roles in proliferation and migration to include additional metabolic regulation. Given the established link between c-MET and PD-L1 induction, these results suggest a convergence of metabolic and immune escape mechanisms. Combined inhibition of c-MET and GLS-1, potentially in conjunction with immune checkpoint blockade, may therefore represent a promising therapeutic strategy for advanced or metastatic HNSCC.

## Supplementary Information

Below is the link to the electronic supplementary material.

Supplementary file 1 Gene expression by MET status in TCGA-HNSC patients. Comparison of gene expression levels between MET-high (n = 261) and MET-low (n = 261) tumors stratified by median MET expression. Analyzed genes include GLS1, HGF, SLC1A5 (ASCT2), DUSP6, MYC, FOSL1, and GLS2. Expression values are shown as log2(TPM + 1). Statistical significance was assessed by Mann–Whitney U test. p* < 0.001, p* < 0.01, p* < 0.05, ns = not significant.

Supplementary file 2 Spearman correlation matrix across HGF/c-MET signaling, MAPK/ERK target, and glutamine metabolism genes in TCGA-HNSC (n = 522). Hierarchically clustered heatmap of pairwise Spearman correlation coefficients for 20 genes encompassing the HGF/c-MET axis (MET, HGF), MAPK/ERK kinases (MAPK1, MAPK3), ERK transcriptional targets (DUSP6, ETV1, ETV4, ETV5, SPRY2, SPRY4, FOSL1, MYC, EGR1, CCND1), and glutamine metabolism genes (GLS, GLS2, SLC1A5, SLC7A5, GLUD1, GOT2). Color scale represents Spearman ρ values.

Supplementary file 3.

## Data Availability

The data that support the findings of this study are available from the corresponding author, M.H., upon reasonable request.
