# Supplementary material for: HGF/c-MET signaling induces glutamine metabolism in MET-amplified head and neck squamous cell carcinoma via MAPK/ERK-dependent induction of GLS-1
Source: Med Oncol. 2026 Sep 15;43(10):287. doi: 10.1007/s12032-026-03409-0 (PMC13578087; doi:10.1007/s12032-026-03409-0)
Supplement: Supplementary file 1 [file 12032_2026_3409_MOESM1_ESM.pdf]

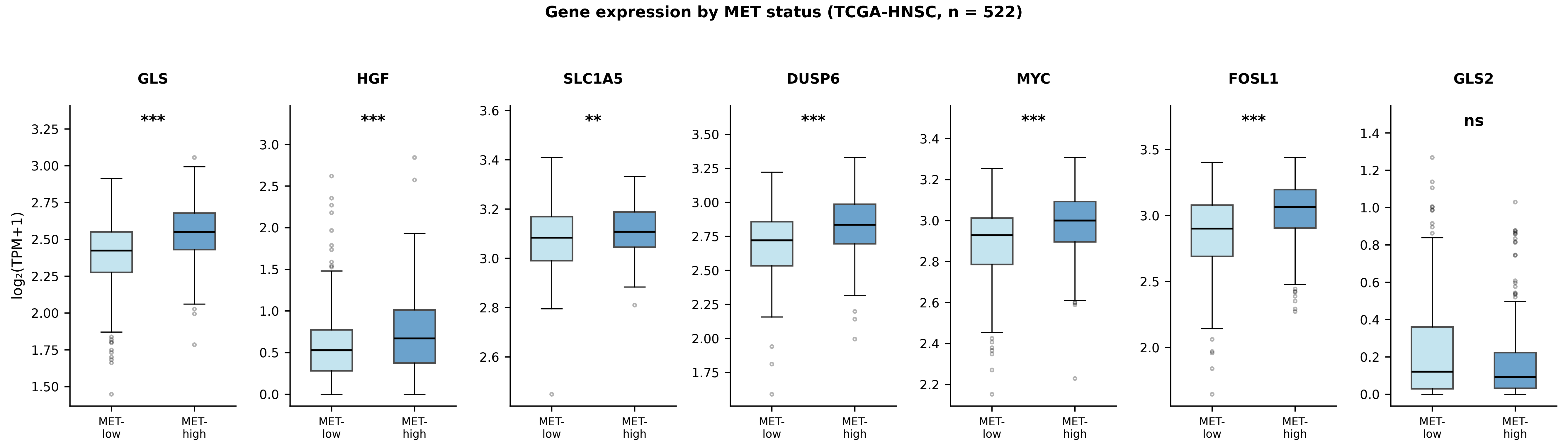
Gene expression by MET status (TCGA-HNSC, n = 522)
GLS
HGF
SLC1A5
DUSP6
MYC
FOSL1
GLS2
***
***
**
***
***
***
ns
log₂(TPM+1)
MET-low
MET-high
