# Supplementary material for: HGF/c-MET signaling induces glutamine metabolism in MET-amplified head and neck squamous cell carcinoma via MAPK/ERK-dependent induction of GLS-1
Source: Med Oncol. 2026 Sep 15;43(10):287. doi: 10.1007/s12032-026-03409-0 (PMC13578087; doi:10.1007/s12032-026-03409-0)

TCGA-HNSC (n = 522): Spearman correlation matrix
HGF/c-MET axis · MAPK/ERK targets · Glutamine metabolism

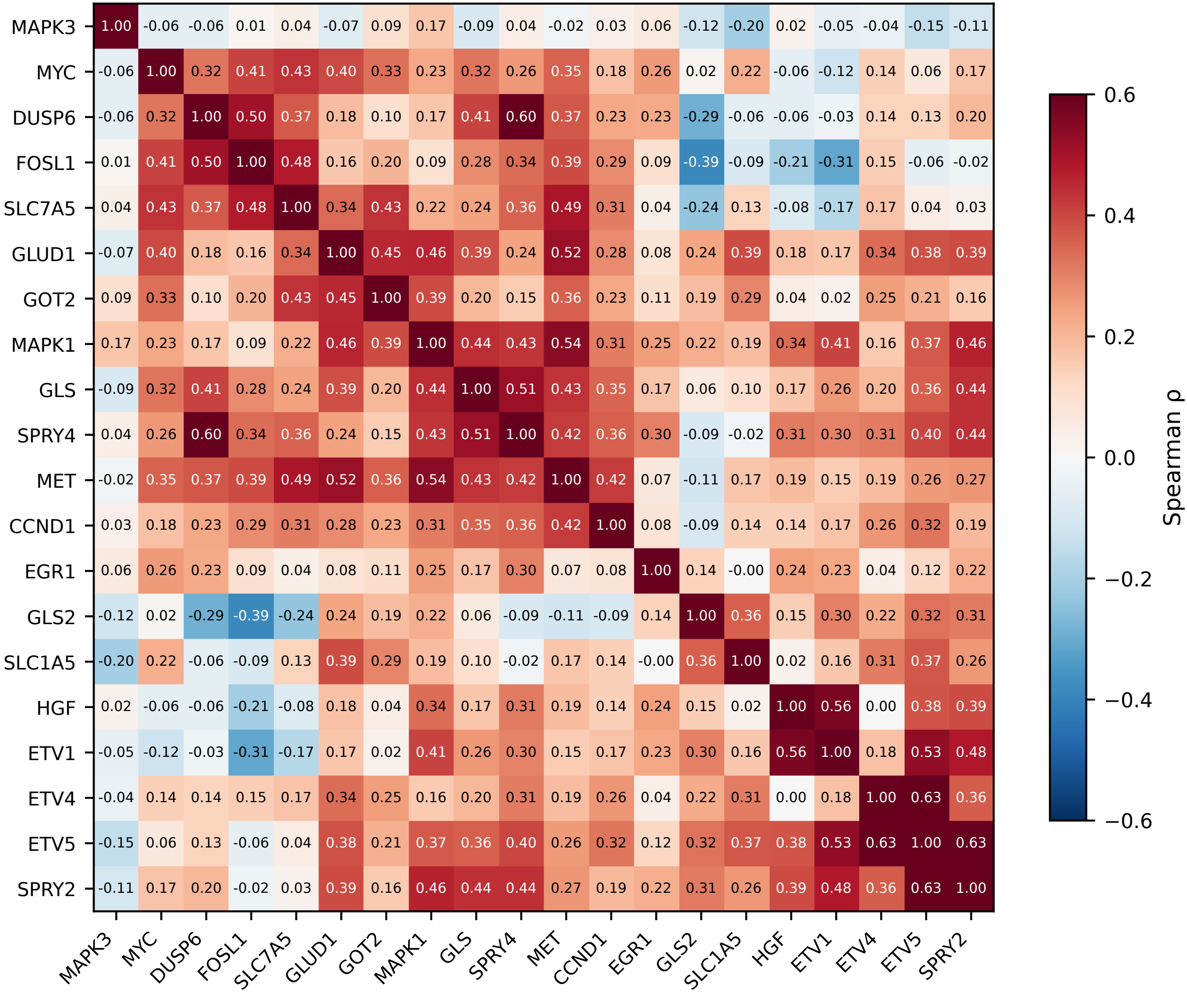

Supplement: Supplementary file 2 [file 12032_2026_3409_MOESM2_ESM.pdf]
