# Supplementary material for: HGF/c-MET signaling induces glutamine metabolism in MET-amplified head and neck squamous cell carcinoma via MAPK/ERK-dependent induction of GLS-1
Source: Med Oncol. 2026 Sep 15;43(10):287. doi: 10.1007/s12032-026-03409-0 (PMC13578087; doi:10.1007/s12032-026-03409-0)

HGF/c-MET Signaling Induces Glutamine Metabolism in MET-Amplified Head and Neck Squamous Cell Carcinoma via MAPK/ERK-Dependent Induction of GLS-1

Original Blots

Fig. 1B GLS-1, ß-actin

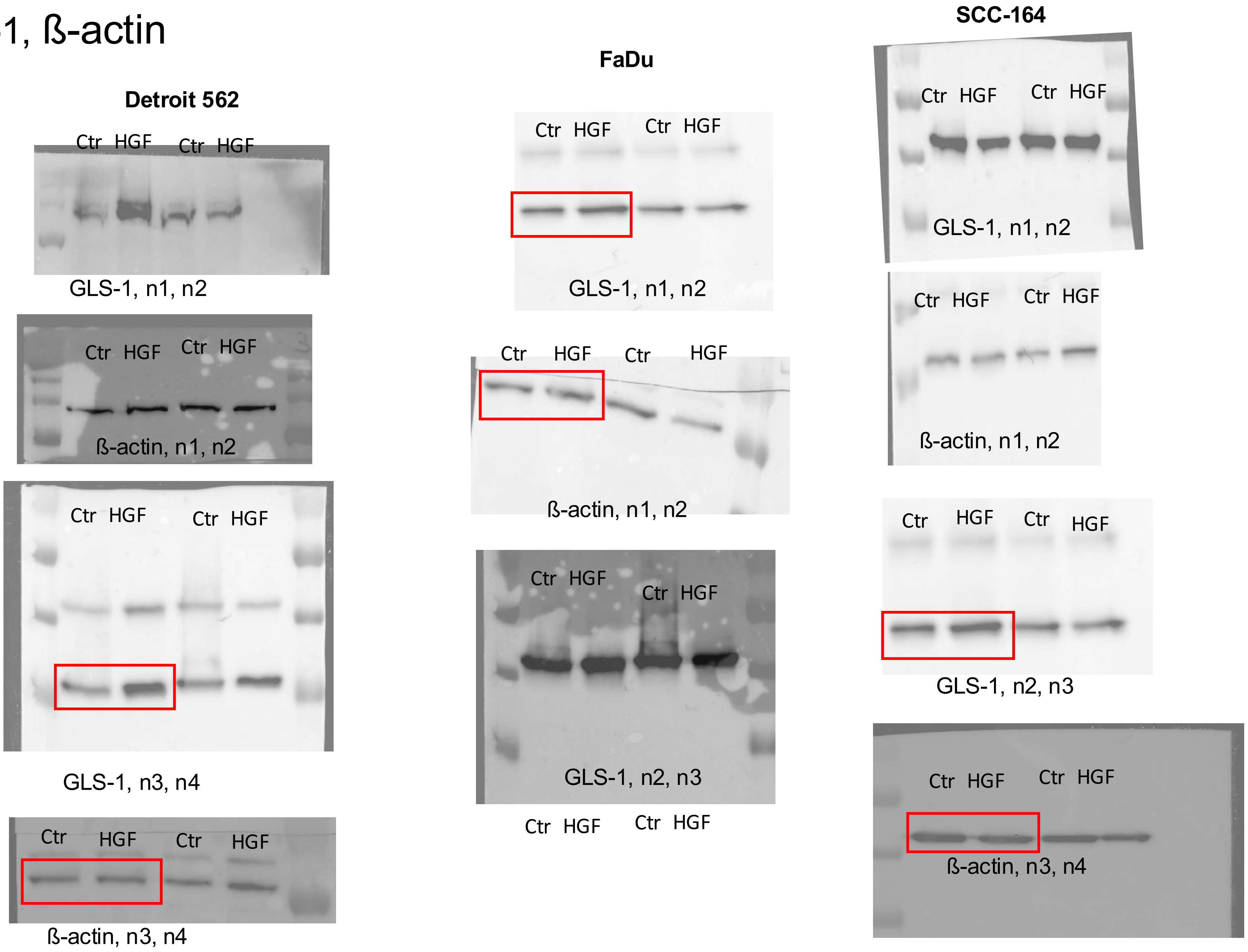

Fig. 1D GLS-1, ß-actin

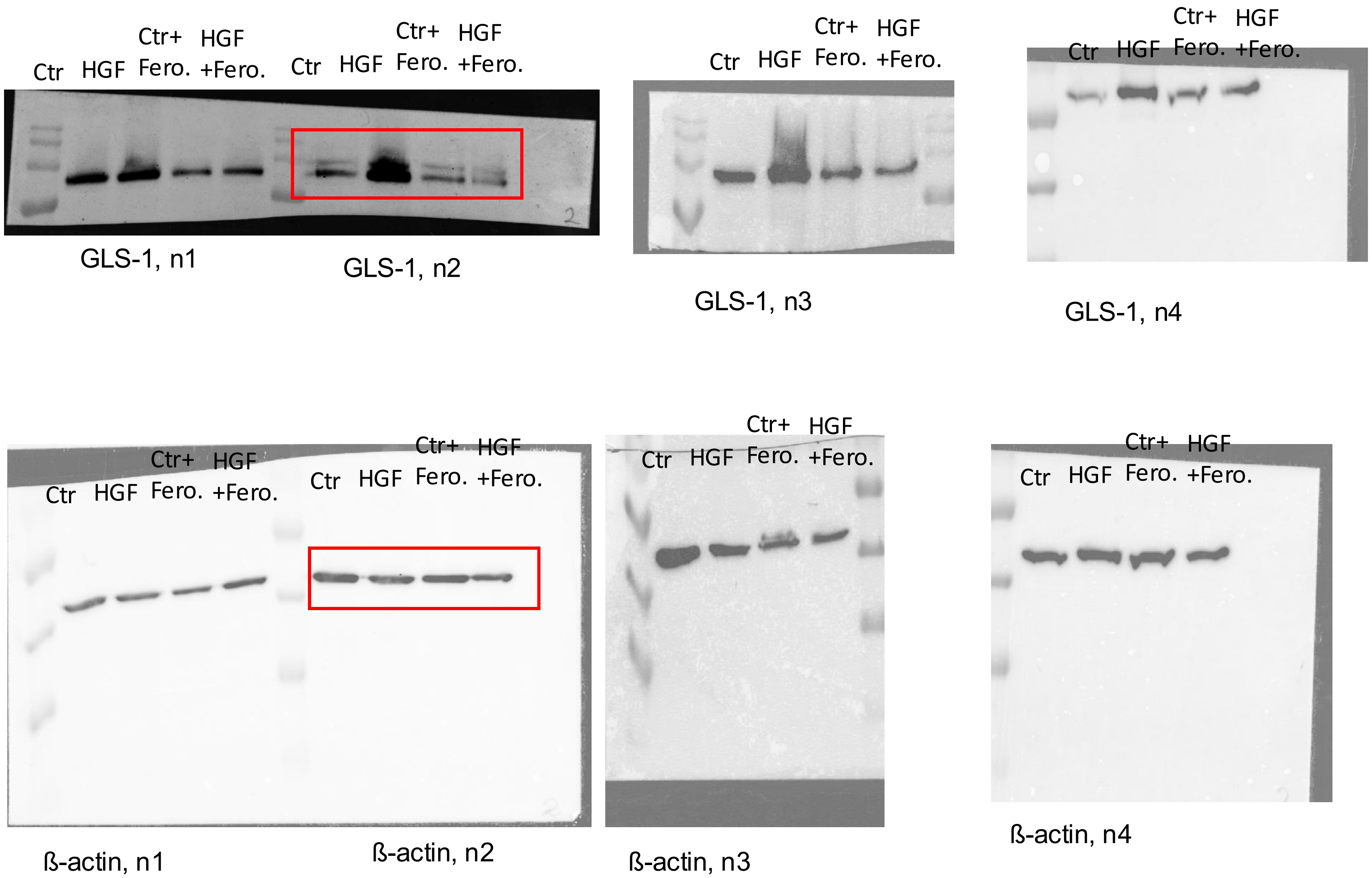

Fig. 2B p-ERK,ERK,

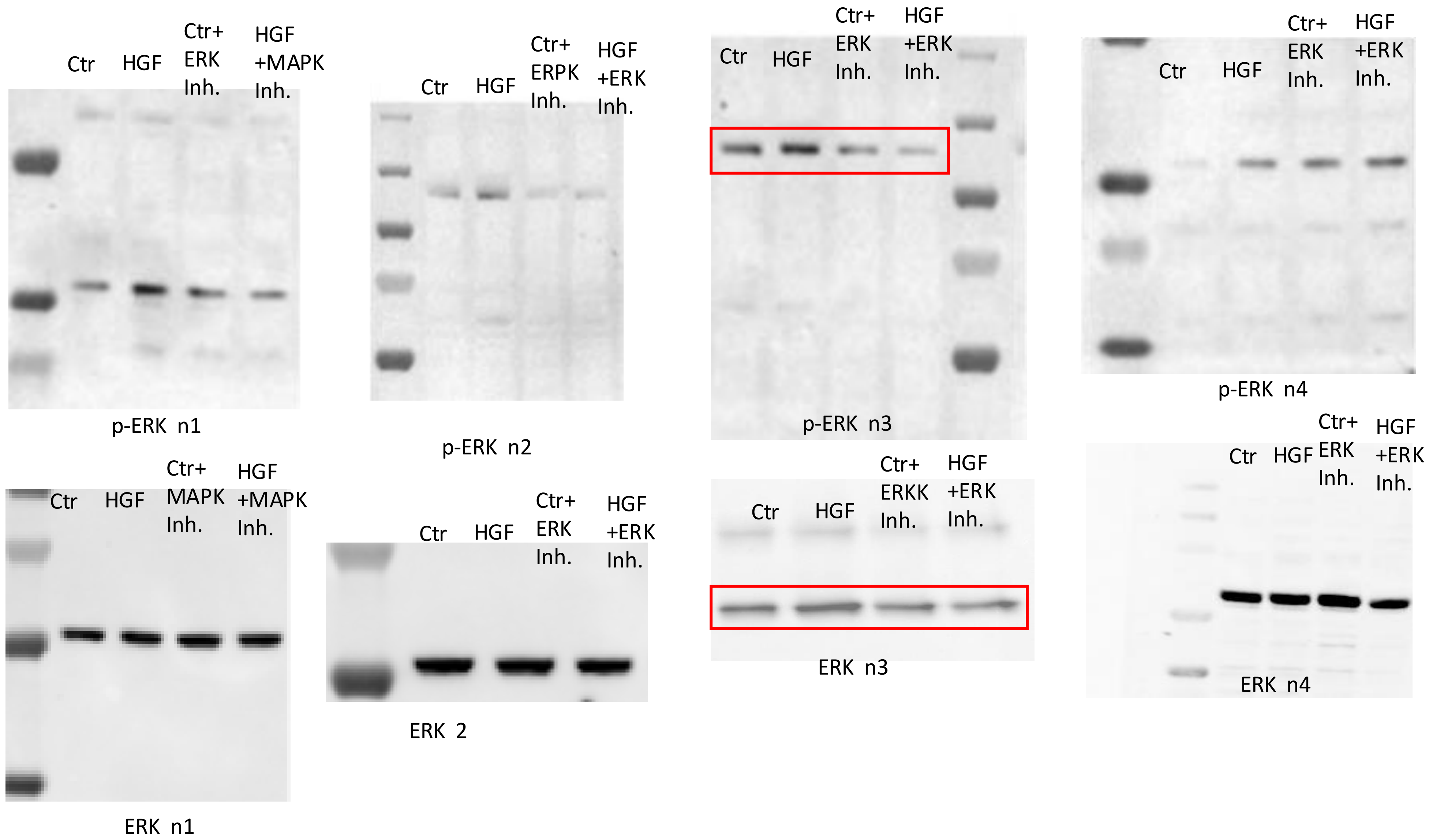

Fig. 2B GLS-1, ß-actin

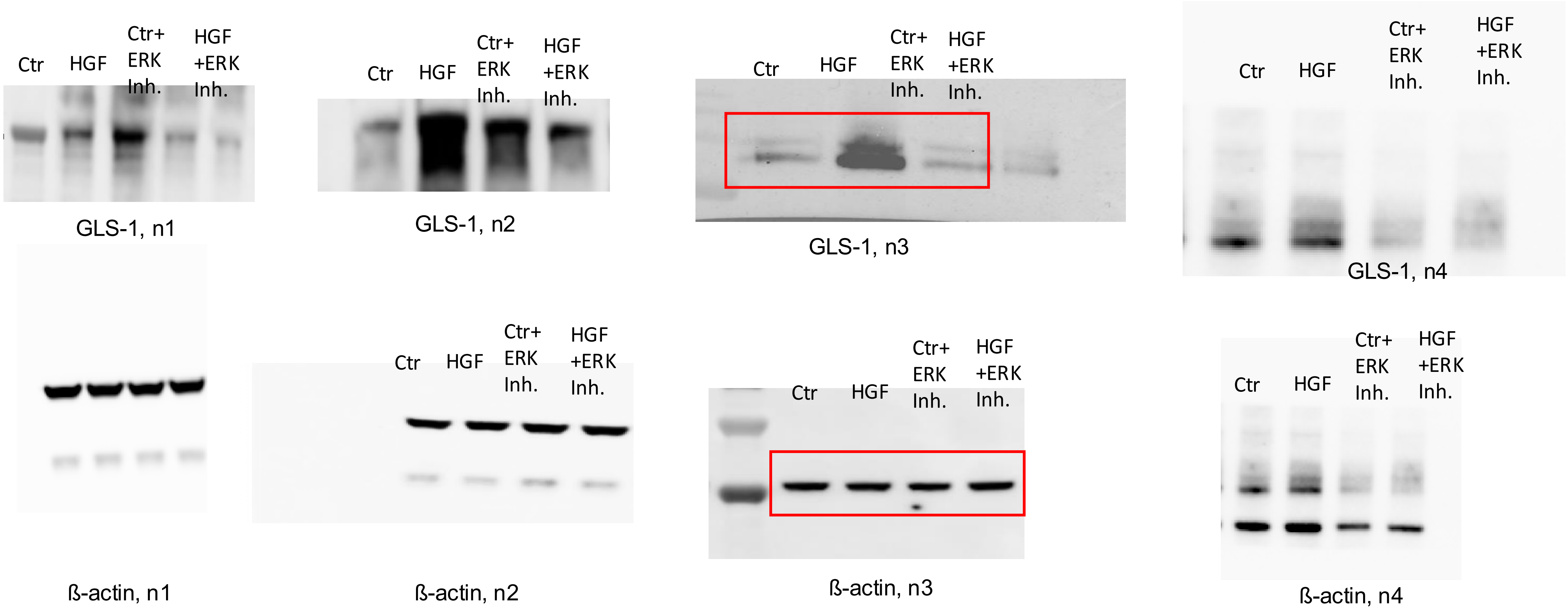

Fig. 2G Ki-67, ß-actin

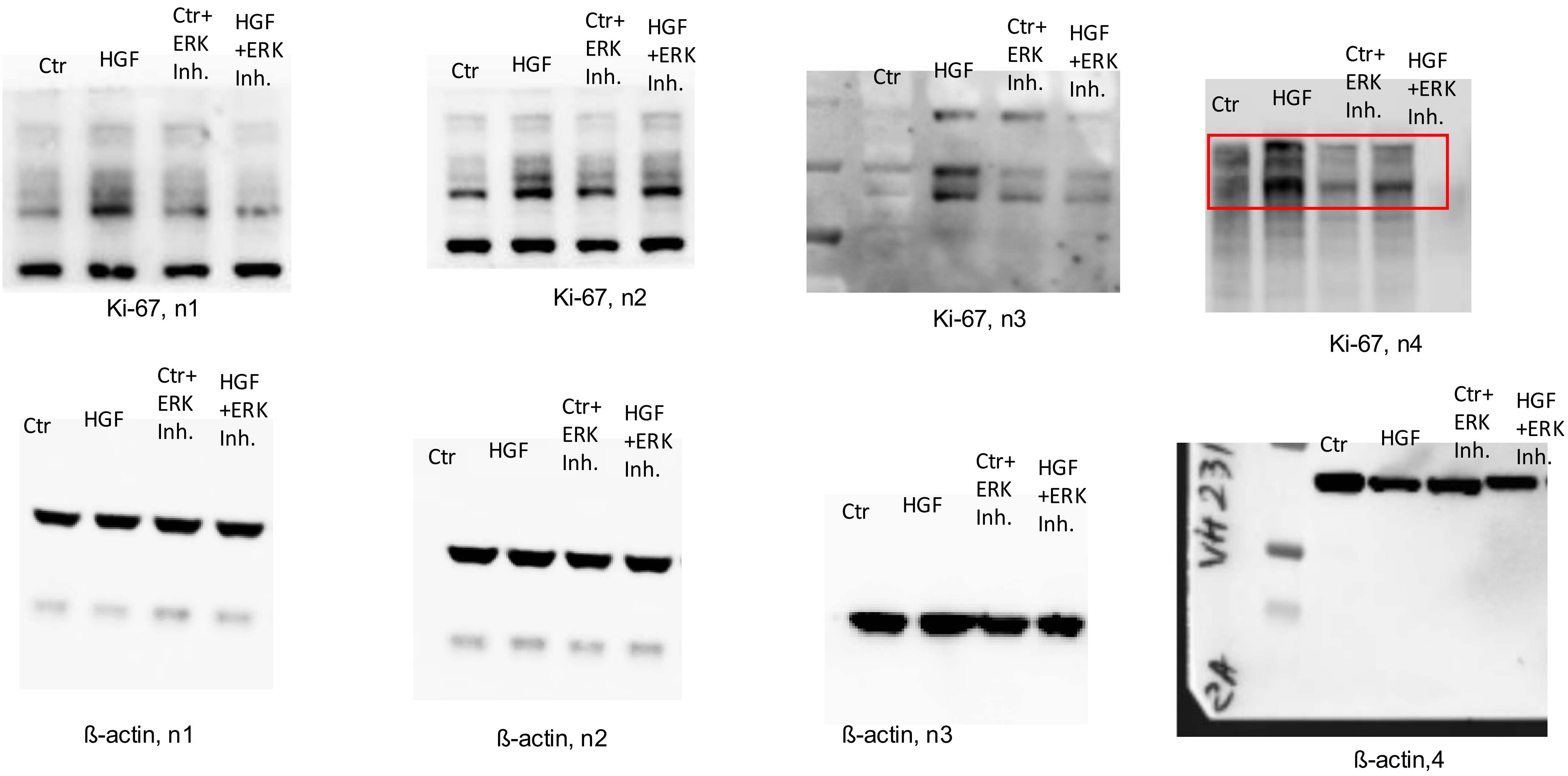

Fig. 2G p-FAK, FAK

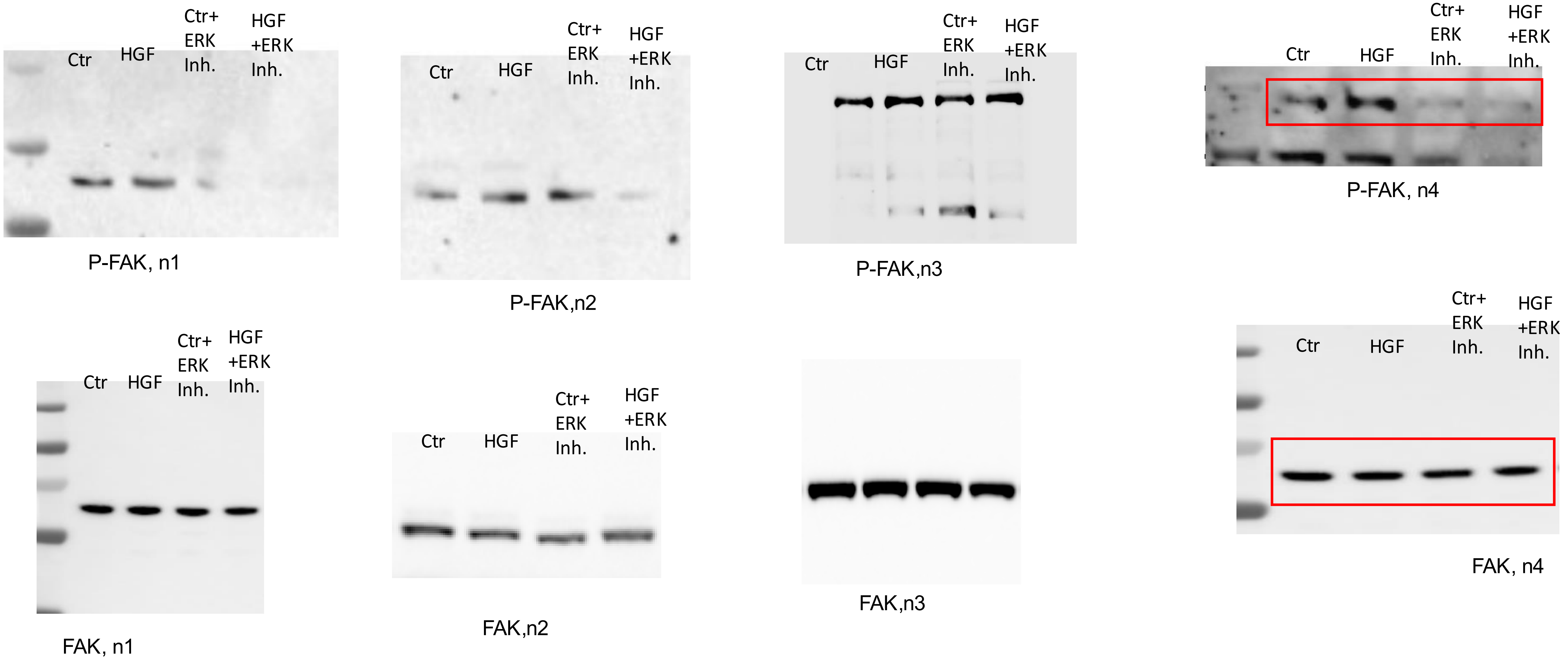

Fig. 3A GLS-1, ß-actin

Detroit 562

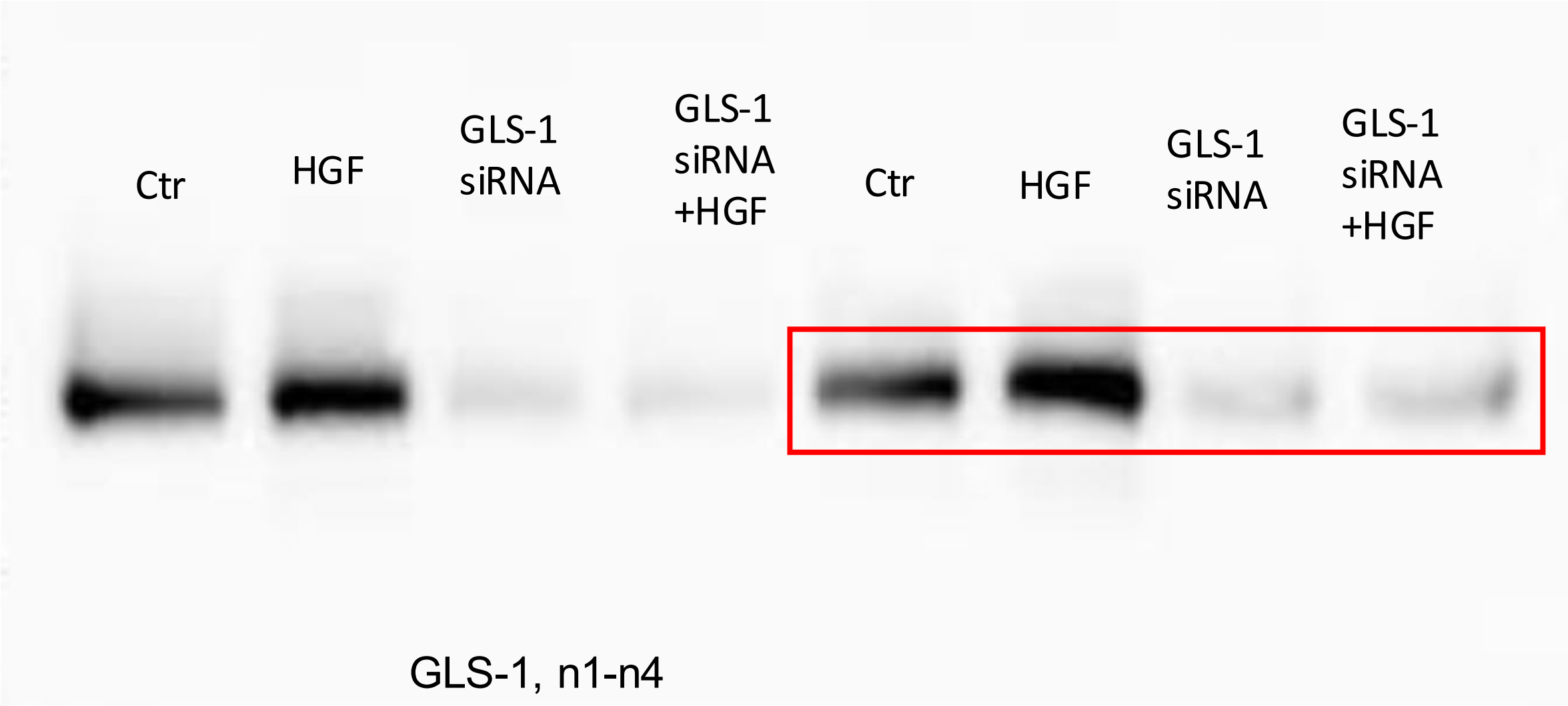

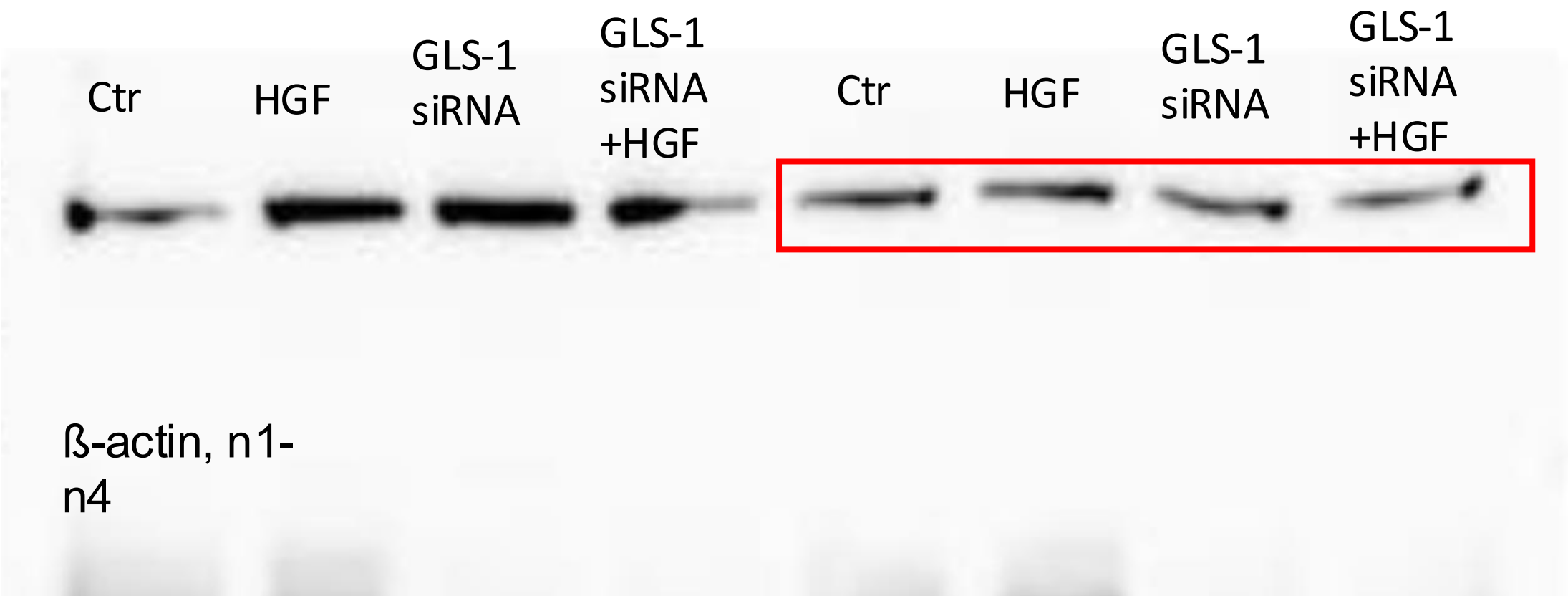

Supplement: Supplementary file 3 [file 12032_2026_3409_MOESM3_ESM.pdf]
